# Duplicité de l’urètre de type IIA2 en “Y”

**DOI:** 10.11604/pamj.2017.27.254.12065

**Published:** 2017-08-04

**Authors:** Mohammed Alae Touzani, Othmane Yddoussalah

**Affiliations:** 1 Université Mohammed V, Faculté de Médecine et de Pharmacie de Rabat, Hopital Ibn Sina, Service d’Urologie B, Rabat, Maroc

**Keywords:** Malformation, urêtre, duplicité, effmann, Lebowitz, Malformation, urethra, duplicity, Effmann and Lebowitz

## Abstract

Urethral duplications are extremely rare congenital malformations. The most used classification is that of Effmann and Lebowitz, describing 6 types of urethral duplications. The under type IIA2-Y is one of the most rare duplications corresponding to duplicated urethra originating from the bladder neck and extending toward the ectopic perineal or anal insertion. We here report the case of a 32 year patient, with no previous personal history, suffering from urinary leakage during and after urination since childhood. Clinical examination showed a bottleneck at the level to the perineum initially suggesting urethral fistula. However, the absence of fistula-associated urinary disorders as well as urinary leakage since childhood refuted this assumption. The patient underwent Micturating Retrograde Urethrocistography after perineal orifice catheterization confirming well systematized tract. Surgical exploration was based on excision of the duplicate urethra after hydrophilic catheterization by inserting hydrophilic guidewire up to its anastomosis at the level of the prostate. The examination of the surgical specimen confirmed the diagnosis of supernumerary urethra.

## Image en médecine

Les duplicités urétrales sont des malformations congénitales extrêmement rares. La classification la plus utilisée est celle d'Effmann et Lebowitz décrivant 6 types, l'un des plus rare étant le sous type IIA2 en 'Y' qui correspond à un trajet de l'urètre dupliqué du col vésical vers un abouchement ectopique périnéal ou anal. Nous rapportons ici le cas d'un patient de 32 ans, sans antécédents, qui présente depuis l'enfance une notion de fuites urinaires lors de la miction et en post-mictionnel. L'examen clinique à mis en évidence un pertuis au niveau périnéal évoquant initialement une fistule urétrale. Cependant, l'absence de troubles urinaires associés ainsi que la notion de fuite depuis l'enfance sont des arguments contre cette hypothèse. Une UCRM à été pratiquée après cathétérisation de l'orifice périnéal confirmant la présence d'un trajet bien systématisé. L'exploration chirurgicale a consisté en l'exérèse de l'urètre dupliqué après cathétérisation par un guide hydrophile jusqu'à son abouchement au niveau prostatique. L'étude de la pièce opératoire a confirmé la nature histologique correspondant à un urètre surnuméraire.

**Figure 1:**
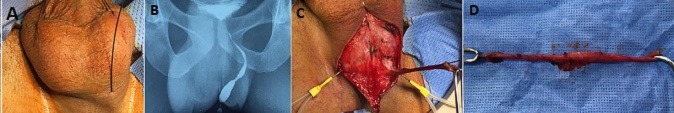
(A) orifice périnéal cathétérisé; (B) opacification; (C) aspect per-opératoire-dissection; (D) urètre surnuméraire

